# B cell lymphoma 6A regulates immune development and function in zebrafish

**DOI:** 10.3389/fcimb.2022.887278

**Published:** 2022-10-28

**Authors:** Farooq L. J. Almohaisen, Somayyeh Heidary, Mohamed L. Sobah, Alister C. Ward, Clifford Liongue

**Affiliations:** ^1^ School of Medicine, Deakin University, Geelong, VIC, Australia; ^2^ Department of Medical Laboratory Technology, Southern Technical University, Basra, Iraq; ^3^ Institute for Mental and Physical Health and Clinical Translation, Deakin University, Geelong, VIC, Australia

**Keywords:** BCL6A, macrophage, immunity, zebrafish, lymphocyte

## Abstract

BCL6A is a transcriptional repressor implicated in the development and survival of B and T lymphoctyes, which is also highly expressed in many non-Hodgkin’s lymphomas, such as diffuse large B cell lymphoma and follicular lymphoma. Roles in other cell types, including macrophages and non-hematopoietic cells, have also been suggested but require further investigation. This study sought to identify and characterize zebrafish BCL6A and investigate its role in immune cell development and function, with a focus on early macrophages. Bioinformatics analysis identified a homologue for BCL6A (*bcl6aa*), as well as an additional fish-specific duplicate (*bcl6ab*) and a homologue for the closely-related BCL6B (*bcl6b*). The human BCL6A and zebrafish Bcl6aa proteins were highly conserved across the constituent BTB/POZ, PEST and zinc finger domains. Expression of *bcl6aa* during early zebrafish embryogenesis was observed in the lateral plate mesoderm, a site of early myeloid cell development, with later expression seen in the brain, eye and thymus. Homozygous *bcl6aa* mutants developed normally until around 14 days post fertilization (dpf), after which their subsequent growth and maturation was severely impacted along with their relative survival, with heterozygous *bcl6aa* mutants showing an intermediate phenotype. Analysis of immune cell development revealed significantly decreased lymphoid and macrophage cells in both homozygous and heterozygous *bcl6aa* mutants, being exacerbated in homozygous mutants. In contrast, the number of neutrophils was unaffected. Only the homozygous *bcl6aa* mutants showed decreased macrophage mobility in response to wounding and reduced ability to contain bacterial infection. Collectively, this suggests strong conservation of BCL6A across evolution, including a role in macrophage biology.

## Introduction

The B cell lymphoma 6A (BCL6A) protein consists of an evolutionarily conserved domain structure, comprising an N-terminal Broad-complex, Tramtrack and Brick-a-brac/Pox virus and Zinc finger family (BTB/POZ) domain, a central PEST domain and a C-terminal zinc finger domain comprising an array of six C_2_H_2_/Krüppel-type zinc fingers (Melnick et al., 2002; Ahmad et al., 2003; Ghetu et al., 2008). It acts as a strong transcriptional repressor, with the zinc finger domain facilitating binding to specific DNA sequences (Dent et al., 1997; Liu et al., 2016) and the BTB/POZ domain enabling recruitment of corepressors, such as SMRT, NCOR, BCOR, MTA3 and CTBP1 (Basso and Dalla-Favera, 2010). BCL6-related proteins are found across a broad range of species. This includes vertebrates, which have been shown to possess distinct but highly-related BCL6A and BCL6B proteins (Okabe et al., 1998), as well as invertebrates, typified by a BCL6-related protein identified in fruit-fly that is referred to as Ken & Barbie (Ken) (Arbouzova et al., 2006).

BCL6A plays a number of critical roles in B and T cell development and function (Wang et al., 2018; Yuan et al., 2022). *Bcl6a* knockout mice exhibited a failure in germinal centre formation in lymph node follicles (Dent et al., 1997; Phan and Dalla-Favera, 2004; Cattoretti et al., 2005) preventing somatic hypermutation and production of high-affinity antibodies (Basso and Dalla-Favera, 2010). This was in part a result of significantly decreased numbers of follicular T helper (Tfh) cells (Nurieva et al., 2009), a lineage in which BCL6A acts as a master regulator (Choi and Crotty, 2021), but also of impaired B cell commitment to the germinal centre B cell lineage (Huang et al., 2014) as well as their subsequent survival (Basso and Dalla-Favera, 2012). *Bcl6a* knockout mice also displayed reduced pre-B cell self-renewal and differentiation in the bone marrow (Duy et al., 2010), with B cell responses to cytokines affected (Basso and Dalla-Favera, 2012). Other T cell subsets were also variably affected, with T helper 2 (Th2) and Th17 cells dramatically increased (Mondal et al., 2010; Choi and Crotty, 2021) and memory T cells decreased (Ichii et al., 2004). *BCL6A* is also considered oncogenic, being highly expressed in many B cell lymphomas such as diffuse large B cell lymphoma (DLBCL) and follicular lymphoma (FL) (Wagner et al., 2011; Green et al., 2014). *Bcl6a* knockout mice also had perturbed dendritic cell development (Ohtsuka et al., 2011), while their macrophages showed altered morphology and defective motility (Pixley et al., 2005) as well as enhanced expression of inflammatory cytokines and chemokines (Toney et al., 2000; Li et al., 2020). *Bcl6a*-deficient mice displayed significantly decreased body weight postnatally (Dent et al., 1997). They also showed poor survival, with most not surviving past 9 weeks, attributed to severe Th2-mediated inflammation of the heart, lungs, liver and spleen (Dent et al., 1997; Yoshida et al., 1999).

Zebrafish is now well established as a model for immune cell development and function. It possesses B, T and NK cells, neutrophils, macrophages, dendritic cells and other immune lineages (Gore et al., 2018). These are generated through conserved developmental processes, which extends to the multiple developmental waves (Bertrand and Traver, 2009), and the associated transcription factors (Kwan and North, 2017). Moreover, their accessibility for genetic and other manipulations, optical transparency and the availability of lineage-specific transgenic lines has enabled new insights into innate immune cell function (Linnerz and Hall, 2020; Rosowski, 2020). This study sought to use zebrafish as a model to further investigate BCL6A function, identifying and characterizing a *BCL6A* homologue that was ablated *via* genome editing to understand the impacts on overall development, growth and survival, including immune cell development and function with a focus on early macrophages.

## Materials and methods

### Bioinformatics

Sequence searches were performed using BLAST on relevant online genetic databases, with Genomescan (Massachusetts Institute of Technology, Cambridge, MA) used to predict protein coding sequences from genomic DNA (Yeh et al., 2001). Sequence analysis, manipulation and assembly were carried out using Sequencher version 4.10.0 (Gene Codes). ClustalX 2.1 (Jeanmougin et al., 1998) was used to generate sequence alignments, from which phylogenetic trees were generated using the Neighbor-Joining algorithm (Saitou and Nei, 1987) with replicates of 1000 and viewed with NJ plot (Perriere and Gouy, 1996) and Treeview 1.6.6 (Page, 1996). Synteny analysis was performed using Ensembl.

### Zebrafish husbandry

Wild-type and *Tg(mpeg1.1::GFP)* (Ellett et al., 2011) zebrafish were maintained using standard husbandry practices (Lawrence, 2007). This included feeding thrice daily with a mixture of live feed (artemia and rotifers) and a dry granulated foodstuff (Otohime Hirame Japan). Embryos were obtained from spawning tanks, and in some cases were injected with either control morpholino (5’-CCTCTTACCTCAGTTACAATTTATA) or anti-sense *bcl6aa* morpholino targeting the intron 2/exon 3 boundary (5’-AGAGCCCACTGTGGAGAAATTATGA) at 0.5 mM. All experiments were approved by the Deakin University Animal Welfare Committee.

### Genome editing

The zebrafish *bcl6aa* gene was targeted using genome editing with CRISPR/Cas9. Embryos were injected with guide RNA (gRNA), designed to a region of exon 3 encoding the BTB/POZ domain using the zifit protocol (Hwang et al., 2013) with the primers 5’-TAGGTCCAGACTGATGGCGTTC and 5’-AAACGAACGCCATCAGTCTGGA, along with Cas9-encoding mRNA and raised to adulthood. Founders were identified with high-resolution melt (HRM) analysis of PCR products with Precision Melt Suremix and Analysis Software (BioRad) (Garritano et al., 2009) using primers spanning the targeted region (5’-CACAGTGGGCTCTTCTACTCTATC and 5’-GGATTGCGAAACCCTCTGG). These fish were outcrossed two times to wild-type fish to remove off-target mutations before in-crossing. Sequence analysis was performed with primers 5’-GCGACCTAAAAAGTTGACTAAAATC and 5’-CCTGGACTTTATGAATCTGTGGC to identify a *bcl6aa* mutant allele (*mdu21*), which was also crossed onto the *Tg(mpeg1.1::GFP)* background.

### Whole-mount *in situ* hybridization

Embryos were dechorionated and fixed in 4% (w/v) paraformaldehyde (PFA) at 4°C prior to WISH with DIG-labeled anti-sense probes, as described (Thisse and Thisse, 2008). Imaging was performed using Olympus MVX10 fluorescence microscope and DP72 camera using Cellsens Dimension 1.6 software, with ImageJ used for quantitation, as required (Abramoff et al., 2004).

### Quantitative real-time reverse-transcription PCR

Total RNA was extracted from whole embryos or juvenile zebrafish using an RNeasy Mini Kit (Qiagen) according to the manufacturer’s protocol for animal tissues. This was subjected to quantitative real-time reverse-transcription PCR with immune cell gene specific primers (*cd4*, *cd8*, *cd79a*, *ighm*, *mpeg1.1*, *mpo*, *nklb*, *nkld* and *tcr*) (Sertori et al., 2022) along with *ccr2* (5’-TGGCAACGCAAAGGCTTTCAGTGA; 5’-TCAGCTAGGGCTAGGTTGAAGAG), *cxcr4b* (5’-CCCATCACAAGCACCACAAG; CGATAGCATCATTTTAGACAACAG), *il1b* (5’-GGACTTCGCAGCACAAAATG; 5’-GTTCACTTCACGCTCTTGGATG), *tgfb1* (5’-AAATAGCAGGTTTGTCCCGC; 5’-CACTTCCAGCCCAGGTCTT) and *tnfa* (5’-GACTGAGGAACAAGTGCTTATGAG; 5’-TGCCCAGTCTGTCTCCTTCTC). Data were normalized to β-actin (*actb*) and fold change calculated using the ΔΔCt method (Livak and Schmittgen, 2001).

### Wounding assay

Wounding assays were performed on 3 dpf embryos (n>20 mixed progeny) by excising the end of the caudal tail fin with a scalpel after anesthesia with 0.1 mg/mL benzocaine (Hall et al., 2007) in a conservative manner as described (Meier et al., 2022), with the number of migrating cells and the number of embryos with migrating cells counted up to 8 h after wounding using fluorescence microscopy.

### Infection assay

Embryos at 4 dpf were injected with 2-5 nl ~ 5×10^9^ CFU/mL *E. coli* expressing GFP (#25922GFP, ATCC) into the venous return, with bacteria visualized by fluorescence microscopy, as described (Basheer et al., 2020).

### Statistics

Statistical analyses were performed using Graph Pad Prism (Version 8) software. To determine the statistical significance of various treatments, the unpaired independent student’s *t* test was employed, with Welch’s correction, where appropriate.

## Results

### Identification and characterization of BCL6-related genes in zebrafish

**Figure 1:**
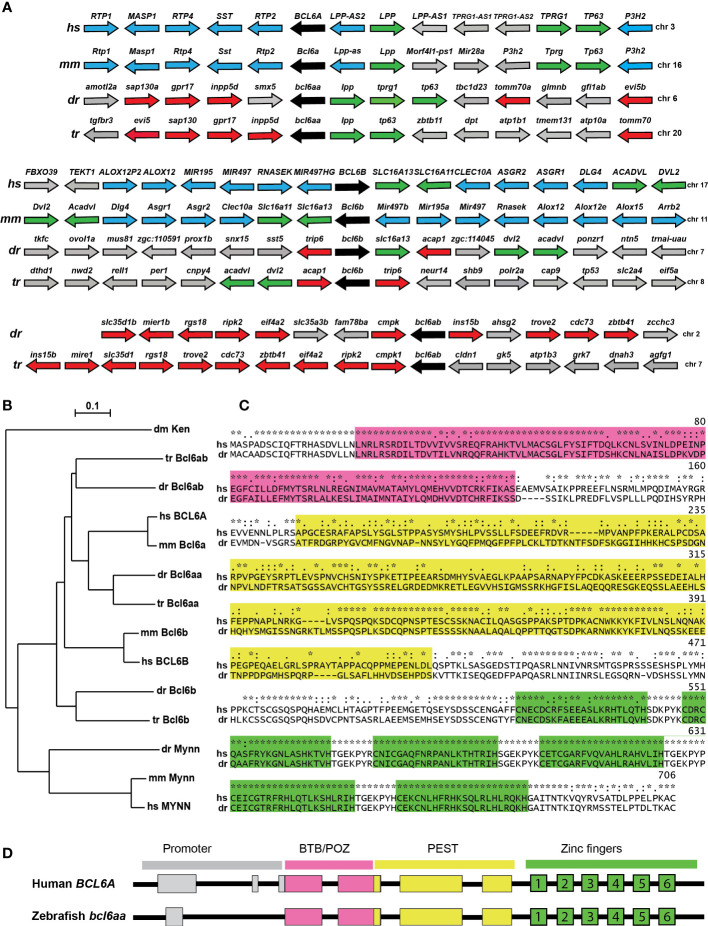
Conservation of BCL6A and related sequences. **(A)** Synteny analysis of *BCL6*-related genes. Arrangement of the gene neighborhood surrounding *BCL6*-related gene loci from human (*Homo sapiens*, hs), mouse (*Mus musculus*, mm), zebrafish (*Danio rerio*, dr) and torafugu (*Takifugu rubripes*, tr). The *BCL6*-related genes are in black, neighboring genes conserved between mammals and fish in green, between mammals in blue and between fish in red, with all other genes in grey. **(B)** Phylogenetic analysis of BCL6-related proteins. The amino acid sequences of fruit-fly Ken and Barbie (Ken) was aligned with the BCL6A and related sequences of human (hs), mouse (mm), zebrafish (dr) and torafugu (tr), and the MYNN-related sequences from human, mouse and torafugu using Clustal W. This was used to construct a phylogenetic tree using the Neighbor-Joining method with 1000 replicates, with bootstrapping values shown. **(C)** Conserved domains in BCL6A proteins. Human BCL6A and zebrafish Bcl6aa were aligned using Clustal X software, with specific domains highlighted (BTB/POZ in pink, PEST in yellow, zinc fingers in green). Conserved residues between the two sequences are indicated (identical *, highly similar: similar.). **(D)** Conserved *BCL6A* gene structure. Schematic diagram of human *BCL6A* and zebrafish *bcl6aa* loci, with exons shown as boxes and introns as lines. Regions corresponding to the promoter (grey) or those encoding the BTB/POZ (pink), PEST (yellow) and zinc finger (green and numbered) domains are indicated.

Bioinformatic analysis identified putative zebrafish homologues for both the *BCL6A* and *BCL6B* genes, as well as an additional related sequence, with all three genes also being present in another teleost fish, torafugu (*Takifugu rubripes*). One of these showed conserved synteny with human and mouse *BCL6A* and their adjacent genes *LPP*, *TPRG1* and *TP63* (**Figure 1A**), with the encoded proteins forming a clade with mammalian BCL6A (**Figure 1B**), and so was designated *bcl6aa*. The fish-specific gene showed conserved synteny across fish genomes, but not with *bcl6aa* or *bcl6b* genes (**Figure 1A**), but the encoded proteins formed a larger clade with the BCL6A sequences (**Figure 1B**), and so was named *bcl6ab*. The final gene showed conserved synteny with human and mouse *BCL6B* and their adjacent *SLC16A13*, *ACADVL* and *DVL2* genes (**Figure 1A**), with the encoded fish and mammalian proteins divergent from the other BCL6 proteins (**Figure 1B**), and was designated *bcl6b*. Collectively, this suggests zebrafish *bcl6aa* and *bcl6b* are functional orthologues of mammalian *BCL6A* and *BCL6B*, respectively, while *bcl6ab* represents a fish-specific duplicate of the *BCL6A* gene.

Alignment of the human BCL6A and zebrafish Bcl6aa proteins confirmed the presence of conserved BTB/POZ, PEST and zinc finger domains, which showed 77%, 35% and 96% identity, respectively (**Figure 1C**). Notably, the latter domain included a stretch of 126 identical amino acids that encompassed the last four of the six C2H2-type zinc fingers. Comparison of the genomic and mRNA (**Figure 1D**) revealed a strongly conserved splicing pattern between human *BCL6A* and zebrafish *bcl6aa* genes across the coding exons, with both also possessing a non-coding exon(s) in the proximal promoter region.

### Expression of zebrafish *bcl6aa*

**Figure 2:**
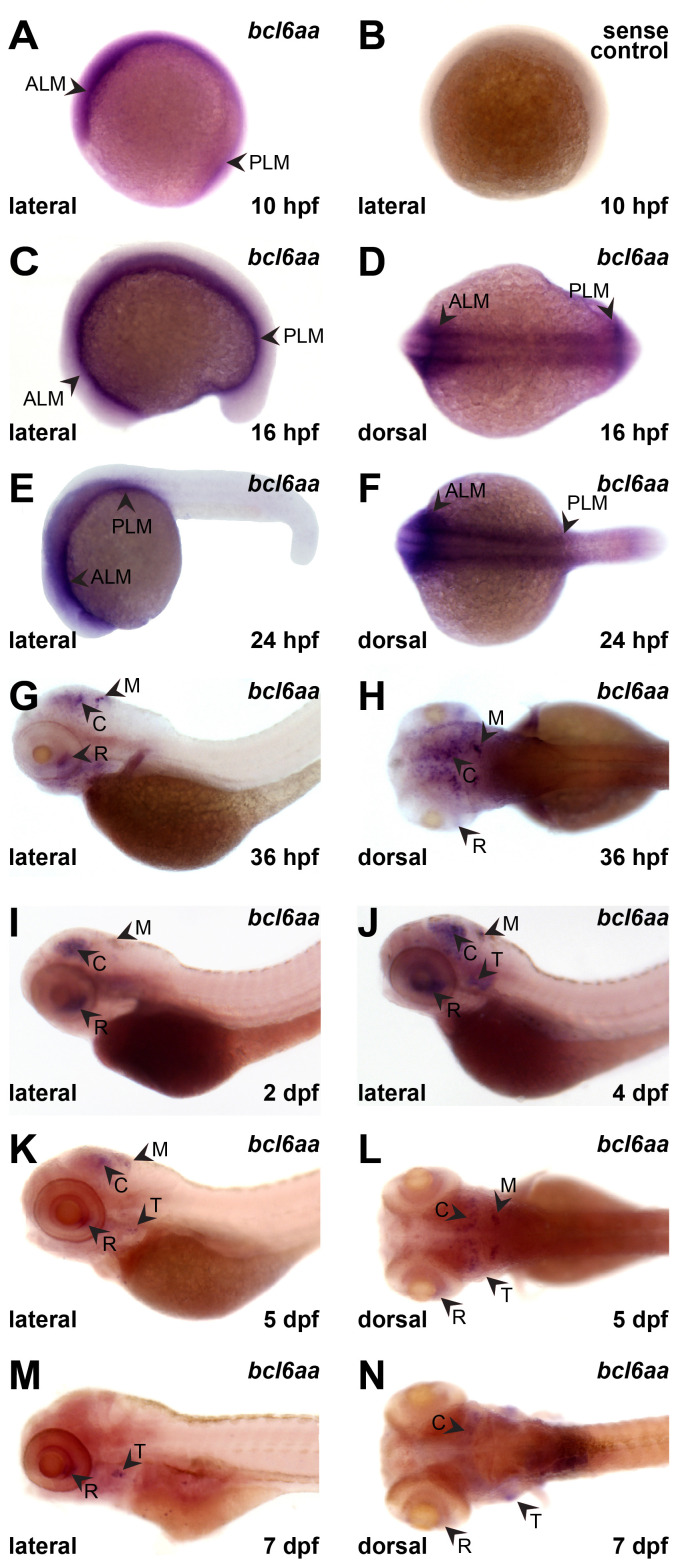
Expression of *bcl6aa* during zebrafish embryogenesis. **(A–L)**. Representative images of wild-type embryos subjected to WISH with anti-sense (*bcl6aa*) and sense (sense control) *bcl6aa* probes as indicated at 10 hpf **(A, B)**, 16 hpf **(C, D)**, 24 hpf **(E, F)**, 36 hpf **(G, H)**, 2 dpf **(I)**, 4 dpf **(J)**, 5 dpf **(K, L)** and 7 dpf **(M, N)**, as viewed laterally or dorsally as labelled. ALM, anterior lateral mesoderm; C, cerebellum; M, medulla; PLM, posterior lateral mesoderm; R, retina; T, thymus.

The embryonic expression pattern of zebrafish *bcl6aa* was investigated by high resolution whole-mount hybridization (WISH) on staged wild-type embryos using an anti-sense *bcl6aa* probe. Expression was observed from 10 hours post-fertilization (hpf) in the anterior lateral mesoderm (ALM) and the posterior lateral mesoderm (PLM), sites of early myeloid cell development (Bertrand and Traver, 2009), which continued until 24 hpf (**Figures 2A, C–F**). From 36 hpf, *bcl6aa* was expressed in the retina, cerebellum and medulla (**Figures 2G, H**) that continued until 7 dpf although declining after 4 dpf (**Figures 2I–N**). From 4 dpf *bcl6aa* expression was also detected in the developing thymus (**Figures 2J–N**), which houses T cell development (Gore et al., 2018). No staining was observed with a control sense *bcl6aa* probe (**Figure 2B** and data not shown).

### Generation and analysis of *bcl6aa* knockout zebrafish

The zebrafish *bcl6aa* gene was mutated using genome editing with CRISPR/Cas9 to target a region of exon 3 encoding the BTB/POZ domain (**Supplementary Figures 1A, B**). Potential founders were identified with high-resolution melt analysis of PCR products spanning the targeted region, with these outcrossed two times to wild-type fish to remove potential off-target mutations before in-crossing. Sequence analysis identified a *bcl6aa* allele (*mdu21*) that harbored a combined large deletion and insertion, predicted to encode a protein that shared just the first 70 amino acids with the wild-type protein, and then encodes 27 amino acids of unrelated sequence before a stop codon is reached (**Supplementary Figure 1C**). Since this represents only part of the BTB/POZ domain and none the PEST or zinc finger domains, it is anticipated that the encoded mutant protein would be non-functional.

**Figure 3:**
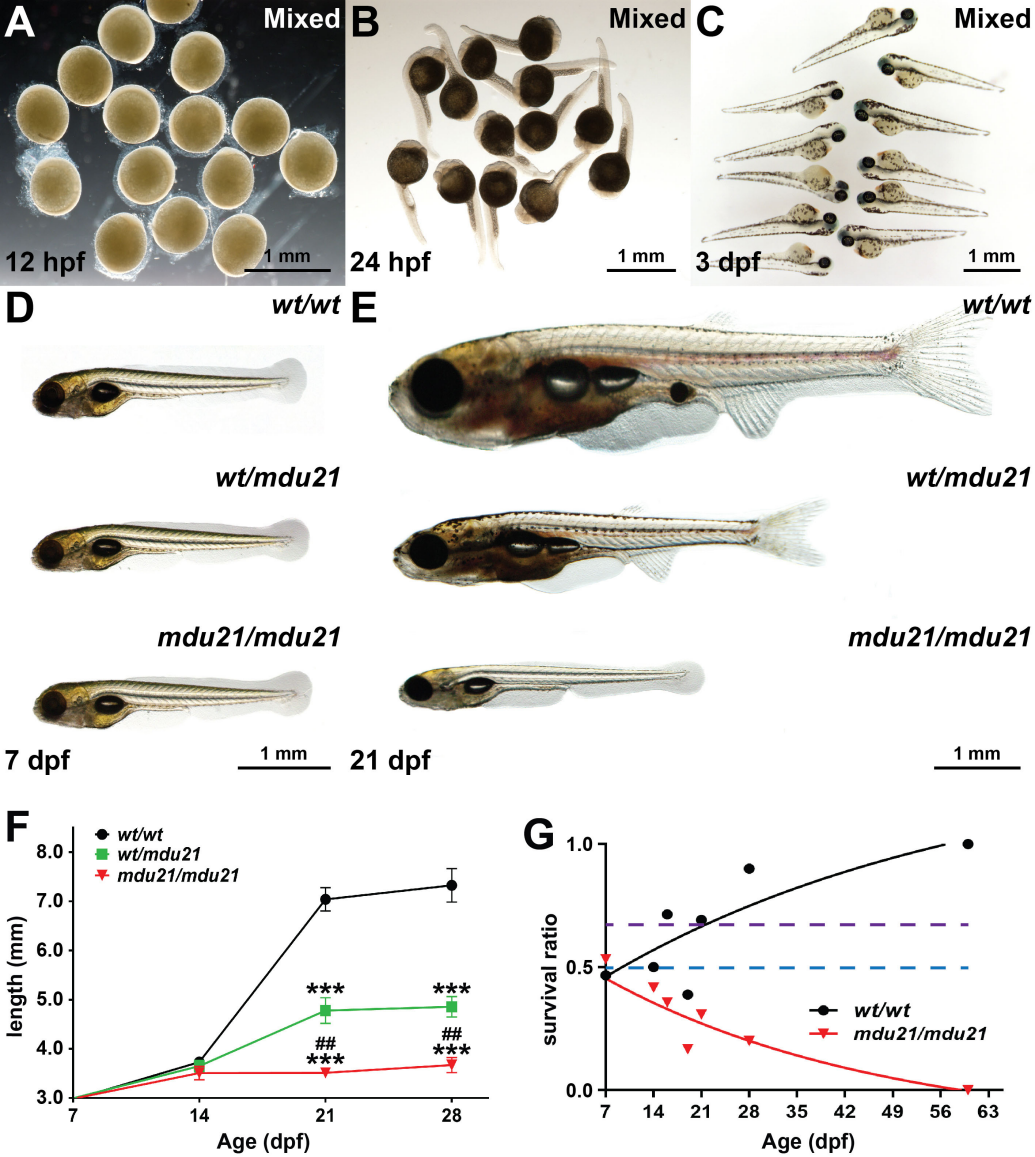
Impact of *bcl6aa* ablation on global development. **(A–E)**. Light microscopy of representative images of mixed progeny (Mixed) derived from *bcl6aa^wt/mdu21^* in-crossing at 12 hpf **(A)**, 24 hpf **(B)** and 3 dpf **(C)** or of individually genotyped *bcl6aa^wt/wt^* (*wt/wt*), *bcl6aa^wt/mdu21^* (*wt/mdu21*) and *bcl6aa^mdu21/mdu21^* (*mdu21/mdu21*) embryos at 7 dpf **(D)** and 21 dpf **(E)**, with 0.5 mm scale bars indicated. **(F)**. Body length of individually genotyped *bcl6aa^wt/wt^* (*wt/wt*), *bcl6aa^wt/mdu21^* (*wt/mdu21*) and *bcl6aa^mdu21/mdu21^* (*mdu21/mdu21*) individuals at the indicated time-points. Shown is the mean ± SEM, with statistical significance relative to *wt/wt* (***p<0.001 and *wt/mdu21* (^##^ *p*<0.01), (n>25). **(G)**. Relative survival of *bcl6aa^wt/wt^* (*wt/wt*) and *bcl6aa^mdu21/mdu21^* (*mdu21/mdu21*) individuals expressed as a ratio relative to *bcl6aa^wt/mdu21^* individuals from n>60 genotypes at each time point. The dotted lines show the expected Mendelian ratio for both *wt/wt* and *mdu21/mdu21* individuals if all genotypes showed equivalent survival (blue) or for *wt/wt* individuals if they showed equivalent survival with *wt/mdu21* in the absence of *mdu21/mdu21* individuals (purple).

The progeny of *bcl6aa^wt/mdu21^* in-crosses were imaged by light microscopy, with no evidence of overt developmental perturbation during embryogenesis observed in mixed groups, which should contain 25% *bcl6aa^mdu21/mdu21^* embryos (**Figures 3A–C**), or in individually genotyped embryos (**Figure 3D** and data not shown). However, this changed dramatically during the juvenile phase, such that by 21 dpf there were large and distinct differences in size that were in roughly Mendelian ratios. Genotyping of individual fish confirmed homozygote *bcl6aa^mdu21/mdu21^* mutants were the smallest, with *bcl6aa^wt/mdu21^* heterozygotes intermediate in size compared to the larger wild-type *bcl6aa^wt/wt^* individuals (**Figure 3E, F**). Additionally, the *bcl6aa^mdu21/mdu21^* mutants showed a clearly under-developed dorsal fin, abdominal fin, tail fin, swim bladder and eye, with the *bcl6aa^wt/mdu21^* mutants again showing an intermediate phenotype.

It was also noted that the proportion of smaller fish decreased over time, with none surviving to adulthood. Genotyping of adult fish confirmed that no *bcl6aa^mdu21/mdu21^* fish were present (data not shown). Close analysis of the relative proportion of *bcl6aa^mdu21/mdu21^* fish at by genotyping across multiple timepoints revealed that while present at an expected Mendelian ratio at 7 dpf, this steadily decreased, with none observed at 60 dpf (**Figure 3G**). The proportion of *bcl6aa^wt/wt^* fish also increased to above the expected Mendelian ratio, indicating that heterozygote fish also had a milder survival defect, although a good proportion survived to adulthood and showed robust fecundity.

### Impact of *bcl6aa* ablation on immune cells

**Figure 4:**
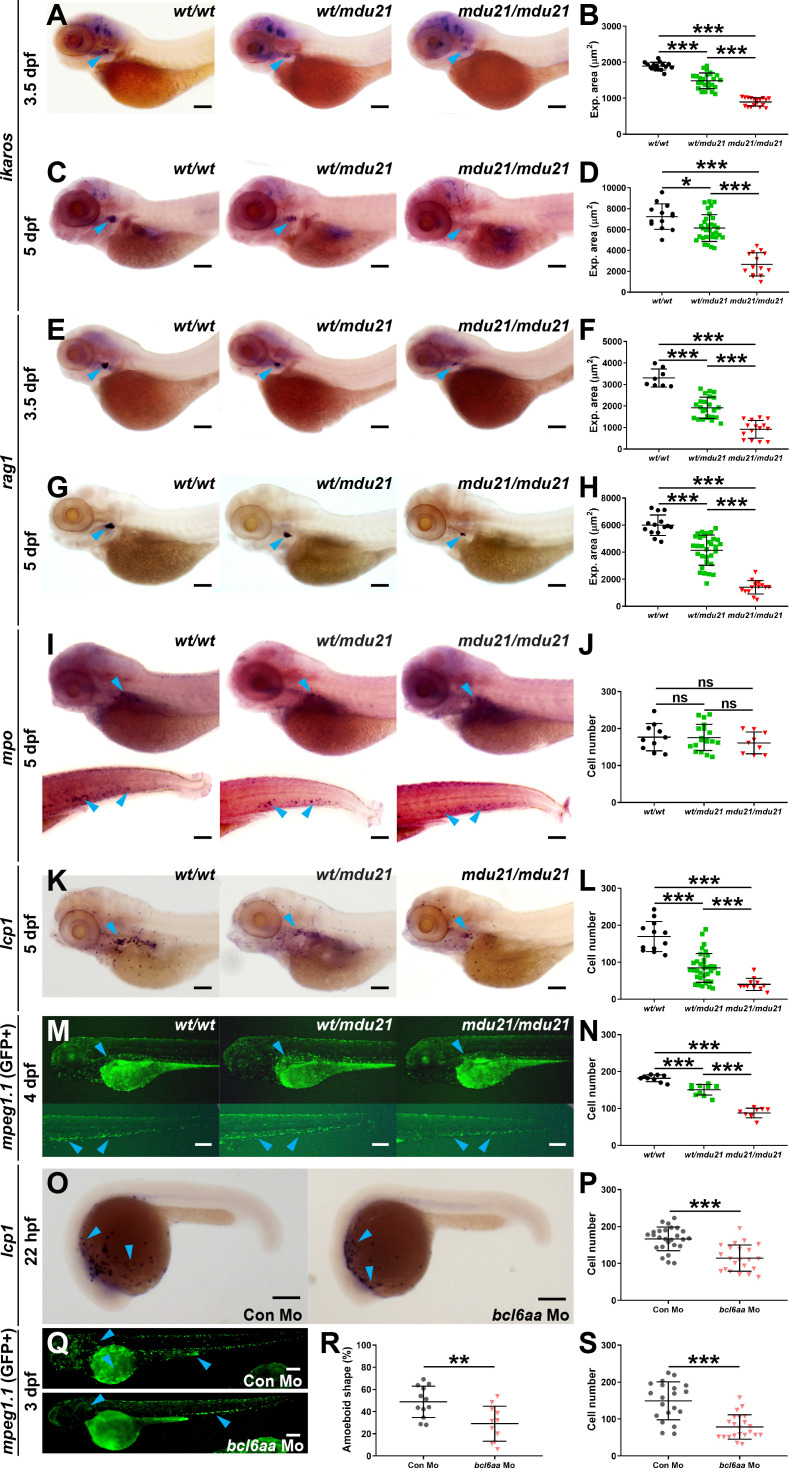
Analysis of lymphoid and myeloid cells in *bcl6aa* mutant zebrafish. **(A, C, E, G, I, K)**. Representative *bcl6aa^wt/wt^* (*wt/wt*), *bcl6aa^wt/mdu21^* (*wt/mdu21*) and *bcl6aa^mdu21/mdu21^* (*mdu21/mdu21*) embryos analyzed by WISH with *ikzf1* at 3.5 dpf **(A)** and 5 dpf **(C)**, *rag1* at 3.5 dpf **(E)** and 5 dpf **(G)**, *mpo* at 5 dpf **(I)** and *lcp1* at 5 dpf **(K)**. **(M)**. Fluorescence imaging of representative *bcl6aa^wt/wt^* (*wt/wt*), *bcl6aa^wt/mdu21^* (*wt/mdu21*) and *bcl6aa^mdu21/mdu21^* (*mdu21/mdu21*) embryos on the *Tg(mpeg1.1:GFP)* background at 4 dpf. **(O)**. Representative wild-type embryos injected with control (Con) or *bcl6aa* morpholino (Mo) as indicated analyzed by WISH with *lcp1* at 22 hpf. **(Q)**. Representative *Tg(mpeg1.1:GFP)* embryos injected with control (Con) or *bcl6aa* morpholino (Mo) subjected to fluorescence microscopy at 3 dpf. Domains of expression are indicated with arrowheads, and scale bars represent 200 μm. **(B, D, F, H, J, L, N, P, R, S)**. Quantification of cell markers, either using expression area **(B, D, F, H)**, number of discrete cells **(J, L, N, P, S)** or the proportion with an amoeboid morphology **(R)**, showing values for individual embryos, as well as mean ± SEM (*p<0.05; **p<0.01; ***p<0.001; ns, not significant; n>30).

**Figure 5:**
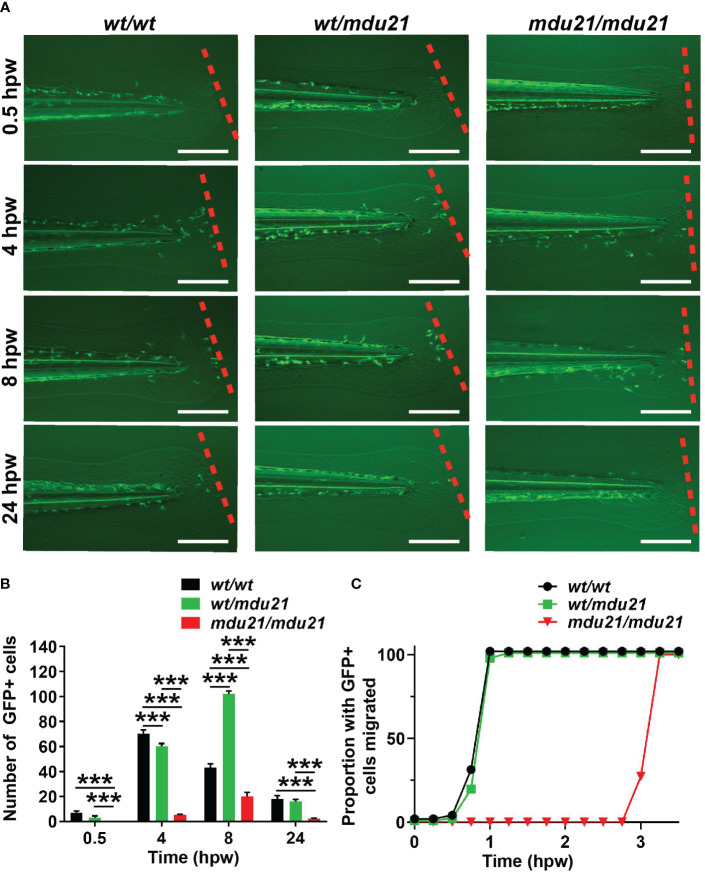
Analysis of macrophage migration in response to injury in *bcl6aa* mutant zebrafish. **(A)** Wounding assay on 4 dpf embryos subjected to injury *via* tail fin transection, showing fluorescence images of representative *bcl6aa^wt/wt^* (*wt/wt*), *bcl6aa^wt/mdu21^* (*wt/mdu21*) and *bcl6aa^mdu21/mdu21^* (*mdu21/mdu21*) embryos on the *Tg(mpeg1.1:GFP)* background, as determined by retrospective genotyping, at the times indicated, with the dotted line showing the wounding site. Scale bars represent 200 μm. **(B)** Quantitation of the total number of GFP+ macrophage migrated at the indicated timepoint showing mean ± SEM. (***p<0.001; n>20 mixed progeny). **(C)** Cumulative proportion of embryos with at least 1 GFP+ cell migrated assessed at 0.25 h intervals (n>20).

The effect of *bcl6aa* ablation on immune cells was investigated by WISH analysis with specific markers during embryogenesis, before any growth or survival defects were present. Homozygote *bcl6aa^mdu21/mdu21^* embryos showed a significant decrease in expression of *ikzf1*, a marker of T cell progenitors in the developing thymus (Willett et al., 2001), compared to *bcl6aa^wt/wt^* and *bcl6aa^wt/mdu21^* siblings at both 3.5 dpf (**Figures 4A, B**) and 5 dpf (**Figures 4C, D**). Expression of *rag1*, a marker of mature T cells (Willett et al., 1997) was significantly decreased in both *bcl6aa^wt/mdu21^* and *bcl6aa^mdu21/mdu21^* embryos compared to *bcl6aa^wt/wt^* siblings at both 3.5 dpf (**Figures 4E, F**) and 5 dpf (**Figures 4G, H**), but to a much greater extent in *bcl6aa^mdu21/mdu21^* embryos across both timepoints (**Figures 4E–H**). In contrast, no significant difference was observed in the number of cells expressing *mpo*, a marker of neutrophils (Lieschke et al., 2001), in either *bcl6aa^wt/mdu21^* or *bcl6aa^mdu21/mdu21^* compared to *bcl6aa^wt/wt^* embryos at 5 dpf (**Figures 4I, J**). However, the number of cells expressing *lcp1*, a marker of leukocytes including macrophages (Bennett et al., 2001), was significantly decreased in both *bcl6aa^wt/mdu21^* and *bcl6aa^mdu21/mdu21^* compared to *bcl6aa^wt/wt^* embryos, although again the quantity in *bcl6aa^mdu21/mdu21^* embryos was also significantly reduced compared to heterozygotes (**Figures 4K, L**). To facilitate further analysis of macrophages the *bcl6aa^mdu21^* allele was crossed onto the *Tg(mpeg1.1:GFP)* background, in which macrophages are marked with GFP (Wittamer et al., 2011). *Tg(mpeg1.1:GFP) bcl6aa^wt/mdu21^* fish were in-crossed and visualized by fluorescence microscopy that revealed a significant decrease in GFP^+^ cells at 4 dpf in *bcl6aa^mdu21/mdu21^* compared to the *bcl6aa^wt/wt^* and *bcl6aa^wt/mdu21^* embryos (**Figures 4M, N**). To confirm the effects of *bcl6aa* on macrophages, embryos were injected with an anti-sense morpholino targeting the intron 2/exon 3 splice site. This also resulted in a decrease of *lcp1+* cells at 22 hpf in wild-type embryos (**Figures 4O, P**) and in GFP+ cells in *Tg(mpeg1.1:GFP)* embryos at 3 dpf (**Figures 4Q, S**) in comparison to those injected with a control morpholino. Macrophage morphology was also altered in the *bcl6aa* morpholino-injected embryos with a statistically significant decrease in those with an amoeboid morphology (**Figure 4R**).

### Functional analysis of *bcl6aa* mutants

To further understand the effect of *bcl6aa* ablation on macrophages, a wounding assay was performed on the progeny of *Tg(mpeg1.1:GFP) bcl6aa^wt/mdu21^* fish as described (Hall et al., 2007), with individual embryos subsequently imaged at various times, after which they were genotyped. This revealed that in *bcl6aa^wt/wt^* embryos GFP+ cells peaked at the wound at 4 hours post wounding (hpw) before decreasing at later time points (**Figures 5A, B**). In both *bcl6aa^wt/mdu21^* and *bcl6aa^mdu21/mdu21^* embryos, GFP+ cells peaked at 8 hpw, however, the numbers observed in *bcl6aa^wt/mdu21^* embryos were equivalent to or exceeded those of *bcl6aa^wt/wt^* embryos, whereas they were significantly reduced in *bcl6aa^mdu21/mdu21^* embryos. When normalized to total GFP+ cells, the number of GFP+ cells remained reduced to a statistically significant level at both 4 and 24 hpw (data not shown). The experiment was repeated but examining embryos at 0.25 h intervals until the first GFP+ cell reached the wound site, after which embryos were genotyped. For almost all *bcl6aa^wt/wt^* and *bcl6aa^wt/mdu21^* embryos this occurred by 1 hpw, but for *bcl6aa^mdu21/mdu21^* embryos this was around 3 hpw (**Figure 5C**).

**Figure 6:**
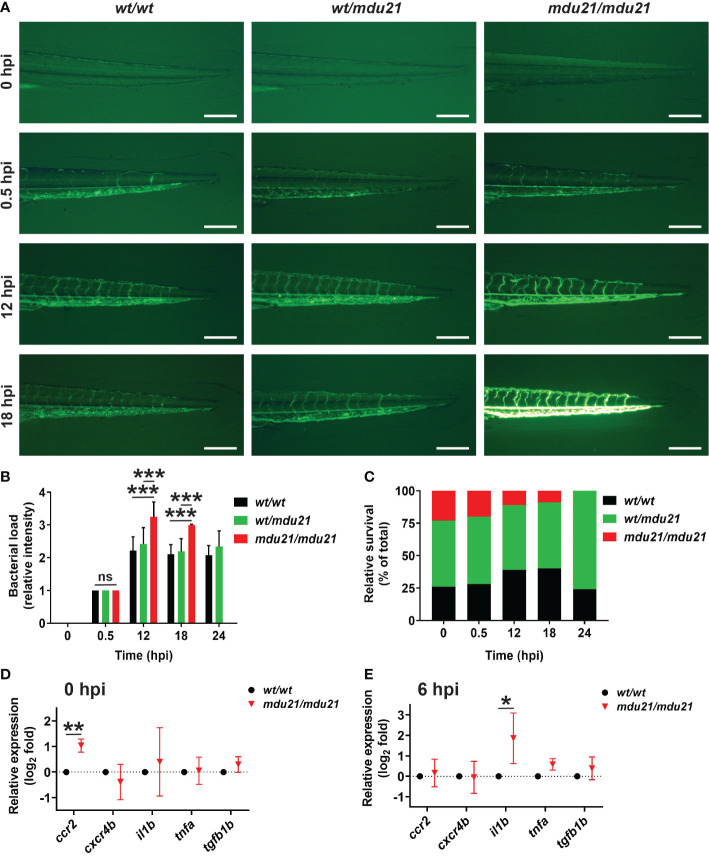
Analysis of bacterial infection in *bcl6aa* mutant zebrafish. **(A)** Infection of 4 dpf embryos with GFP+ *E coli* showing representative *bcl6aa^wt/wt^* (*wt/wt*), *bcl6aa^wt/mdu21^* (*wt/mdu21*) and *bcl6aa^mdu21/mdu21^* (*mdu21/mdu21*) embryos at the indicated timepoints. Scale bars represent 200 μm. **(B)** Bacterial load intensity was assessed on a 5 point scale (0-4) for *bcl6aa^wt/wt^* (*wt/wt*), *bcl6aa^wt/mdu21^* (*wt/mdu21*) and *bcl6aa^mdu21/mdu21^* (*mdu21/mdu21*) embryos at each timepoint relative to 0.5 hpf being 1, showing mean ± SEM (***p<0.001; ns: not significant; n≥50 total at each timepoint). **(C)** Relative survival of *bcl6aa^wt/wt^* (*wt/wt*), *bcl6aa^wt/mdu21^* (*wt/mdu21*) and *bcl6aa^mdu21/mdu21^* (*mdu21/mdu21*) embryos at the indicated timepoints (n=100 total at each timepoint). **(D, E)** Analysis of the indicated inflammatory gene markers in homozygous *bcl6aa^wt/wt^* (*wt/wt*) and *bcl6aa^mdu21/mdu21^* (*mdu21/mdu21*) individuals at 0 hpi **(D)** and 6 hpi **(E)** using qRT^2^-PCR with data normalized relative to *actb* and represented as relative fold change compared to wild-type, with mean and SD shown and statistical significance indicated (*p<0.05, **p<0.01, n=4).

The *bcl6aa* mutants were next investigated in a bacterial infection assay (Basheer et al., 2020). The progeny of *bcl6aa^wt/mdu21^* in-crosses were subjected to injection with GFP+ *E. coli* at 4 dpf. Surviving embryos were imaged by fluorescent microscopy until 24 hour post infection (hpi) and subsequently genotyped, with the fluorescence intensity used as an indicator of bacterial load. The *bcl6aa^wt/wt^*, *bcl6aa^wt/mdu21^* and *bcl6aa^mdu21/mdu21^* embryos showed no difference in fluorescence at 0.5 hpi (**Figures 6A, B**). However, at 12 and 18 hpi the fluorescence intensity was increased in *bcl6aa^mdu21/mdu21^* compared to *bcl6aa^wt/wt^* and *bcl6aa^wt/mdu21^* embryos. No difference in mortality was observed in the injected embryos at 0.5 hpi, but from 12 hpi decreased survival of *bcl6aa^mdu21/mdu21^* embryos was observed, reaching zero survival at 24 hpi, while *bcl6aa^wt/wt^* and *bcl6aa^wt/mdu21^* showed similar high survival rate (**Figure 6C**) and fluorescence intensity (**Figure 6B** and data not shown). Analysis of a set of inflammatory genes indicated increased basal expression of *ccr2* in *bcl6aa^mdu21/mdu21^* compared to *bcl6aa^wt/wt^* embryos (**Figure 6D**). Infection resulted in increased expression of *il1b* and *tnfb1b* in both *bcl6aa^wt/wt^* and *bcl6aa^mdu21/mdu21^* embryos (data not shown), but *il1b* was significantly enhanced in *bcl6aa^mdu21/mdu21^* compared to *bcl6aa^wt/wt^* embryos (**Figure 6D**).

## Discussion

Mammalian BCL6A, and the closely-related BCL6B, are transcriptional repressors consisting of BTB/POZ, PEST and zinc finger domains (Basso and Dalla-Favera, 2010). BCL6A homologues are highly conserved, with murine BCL6A 95% identical to its human BCL6A counterpart (Fukuda et al., 1995). A zebrafish homologue (*bcl6aa*) was identified on the basis of phylogenetic and syntenic analysis, with the encoded protein displaying >60% identity with human BCL6A, with equivalent BTB/POZ, PEST and zinc finger domains, the latter showing 96% identity, consistent with a *bcl6aa* gene reported from another teleost fish (Ohtani et al., 2006). This high conservation indicates a likely conserved function across vertebrates and particular of target DNA sequences. This study also identified an additional fish-specific gene, *bcl6ab*, most likely a paralogue of *bcl6aa*, one of many teleost genes duplicated as a result of a teleost-specific whole genome duplication (WGD) event (Reams and Roth, 2015), as well as a zebrafish orthologue to the mammalian *BCL6B* gene (*bcl6b*). It will be of interest to investigate whether the *bcl6ab* paralogue has evolved a unique function or shares functions with *bcl6aa* and possibly *bcl6b*.

Mammalian *BCL6A* is strongly expressed in thymocytes from human fetal samples at 21 weeks gestation (Hyjek et al., 2001), and in the fetal mouse thymus at 17 days gestation (Bajalica-Lagercrantz et al., 1998). Zebrafish *bcl6aa* was similarly expressed in the developing thymus during embryogenesis, suggesting a conserved role in early T lymphocyte development across vertebrates. The *bcl6aa* gene was also expressed even earlier in the zebrafish ALM and PLM, which represent sites of primitive hematopoiesis in the zebrafish (Bertrand and Traver, 2009). Expression of *BCL6A* has also been identified in adult peripheral blood leukocytes and lymph nodes in humans (Bajalica-Lagercrantz et al., 1997), and of *Bcl6a* in the adult mouse thymus (Bajalica-Lagercrantz et al., 1998). The pufferfish *bcl6aa* homologue has been previously found to be expressed in adult thymus and kidney, the teleost bone marrow equivalent (Ohtani et al., 2006), while analysis of published single cell sequencing data (Tang et al., 2017) confirms *bcl6aa* is expressed in adult zebrafish T and B cells (data not shown). Collectively, this indicates potential conserved roles for BCL6A in the ongoing development of blood and immune cells in the adult.

The *bcl6aa* gene was also expressed in regions of the developing brain and retina, the latter confirming a previous study (Lee et al., 2013). This is consistent with expression of fruit-fly *ken* at the onset of gastrulation in the cephalic furrow and later in the larval eye-antenna (Arbouzova et al., 2006), with *Bcl6a* expression also seen in the olfactory epithelium of prenatal mice (Bajalica-Lagercrantz et al., 1998). *BCL6A* expression in adult human spinal cord has been described (Bajalica-Lagercrantz et al., 1997), with *Bcl6a* shown to be expressed in the adult mouse cerebral cortex (Bajalica-Lagercrantz et al., 1998) and *bcl6aa* in the adult pufferfish brain and nasal cavity (Ohtani et al., 2006). Human *BCL6A* was also expressed in adult skeletal muscle, thyroid, trachea, ovary and prostate (Bajalica-Lagercrantz et al., 1997), with mouse *Bcl6a* expressed in skeletal muscle (Albagli-Curiel et al., 1998) and pufferfish *bcl6aa* in skeletal muscle, intestine and ovary (Ohtani et al., 2006). Collectively, this may suggest a conserved broader role for *BCL6/ken* genes in non-hematopoietic tissues.

BCL6A has been shown to be a key regulator of B and T cells (Nurieva et al., 2009; Bassil et al., 2014), with specific lineages of both B and T lymphocytes impacted in *Bcl6a* knockout mice (Dent et al., 1997; Cattoretti et al., 2005; Nurieva et al., 2009; Mondal et al., 2010; Huang et al., 2014; Choi and Crotty, 2021). Zebrafish possess both T and B cells (Langenau and Zon, 2005; Hansen and Zapata, 1998; Trede and Zon, 1998), with zebrafish T cells precursors arising during the embryonic definitive wave of hematopoiesis and, as in mammals, generating mature T cells in the thymus (Haire et al., 2000; Bertrand et al., 2007; Seelye et al., 2016). The *bcl6aa^mdu21/mdu21^* mutants displayed a significant decrease in lymphocyte populations at this location during embryogenesis including lymphoid precursors and early T lymphocytes. This finding suggests an essential role of *bcl6aa* in the differentiation and/or survival of early T cells or their progenitors in zebrafish. It was more difficult to study B cells since these arise three weeks post fertilization (Danilova et al., 2000) when survival of *bcl6aa^mdu21/mdu21^* fish was already compromised. Analysis of surviving juveniles at 28 dpf showed multiple lymphocyte populations were reduced, including T, B and NK cells (**Supplementary Figure 2**). However, the significant developmental delay observed in *bcl6aa^mdu21/mdu21^* mutants and the reliance solely on qRT-PCR data means this result needs to be interpreted cautiously. Recent enhancements in husbandry practices have meant that *bcl6aa^mdu21/mdu21^* adults are now available, the analysis of which will provide more definitive understanding of the impacts on lymphocyte homeostasis.

BCL6A has been previously implicated in the development and function of macrophages and dendritic cells (Yamochi et al., 1997; Pantano et al., 2006; Zhang et al., 2014). A significant reduction in macrophages was observed in *bcl6aa^mdu21/mdu21^* embryos, which was also the case in juvenile fish (**Supplementary Figure 2**), and confirmed using morpholino-mediated gene knockdown. Significantly decreased macrophage motility was observed in response to wounding, with macrophages appearing more amoeboid following *bcl6aa* ablation. This is consistent with a study showing inactivation of *Bcl6a* in bone-marrow derived macrophages resulted in reduced macrophage motility, polarization and spreading (Pixley et al., 2005). The *bcl6aa^mdu21/mdu21^* mutants were also found to be less able to control bacterial infection, had elevated *il1b* and reduced survival, in agreement with data from *Bcl6a* knockout mice that showed increased inflammatory gene expression following LPS injection, including *Il1b* (Barish et al., 2010). These effects are likely a consequence of the reduced macrophage number and functionality, since T cells remain in the thymus at this stage of development, and neutrophil numbers are unchanged – although potential functional defects were not examined. Of note, fruit-fly hemocytes, which represent innate immune cells, were also found to be sensitive to the effects of Ken (Arbouzova et al., 2006). This suggests an evolutionarily conserved role for BCL6A/Ken in innate immunity.

*Bcl6a* knockout mice displayed severe growth retardation (Albagli-Curiel et al., 1998; Yoshida et al., 1999), which was also observed in *bcl6aa^mdu21/mdu21^* fish from 14 dpf indicating this represents another common phenotype across vertebrates. This may be mediated *via* a direct role on growth, since strong *Bcl6a* expression has been observed during skeletal muscle differentiation in mice (Bajalica-Lagercrantz et al., 1998). However, the *bcl6aa^mdu21/mdu21^* also displayed a thinner body, consistent with the reduced adipose mass seen in *Bcl6a* knockout mice (LaPensee et al., 2014). This might be due to a direct effect on adipose tissue, as murine *Bcl6a* knockouts have been shown to possess disrupted lipid metabolism (LaPensee et al., 2014). The *bcl6aa^mdu21/mdu21^* fish also displayed reduced survival. A similar phenotype was observed in *Bcl6a* knockout mice, which has been demonstrated to be the result of excessive inflammatory responses leading to profound myocarditis and vasculitis (Dent et al., 1997; Fukuda et al., 1997; Ye et al., 1997; Huang et al., 2014). This severe inflammation could also impact on growth and development indirectly.

Fruit-fly Ken has been found to contribute to the differentiation of photoreceptor (neuronal) cells and cone (non-neuronal) cells during eye development (Wen et al., 2000). Moreover, a previous study showed that *bcl6aa* knockdown in zebrafish embryos resulted in malformation of the optic cup during embryogenesis (Lee et al., 2013). We observed expression of *bcl6aa* in the developing zebrafish eye but no overt eye defects in *bcl6aa^mdu21/mdu21^* mutants, with none reported in *Bcl6a* mutant mice either. More work is required to understand the discrepancies, especially between the zebrafish studies, as well to investigate other aspects of development that are perturbed in *Bcl6a* knockout mice and *ken* mutant flies.

## Data availability statement

The original contributions presented in the study are included in the article/**Supplementary Materials**. Further inquiries can be directed to the corresponding author.

## Ethics statement

The animal study was reviewed and approved by Deakin University Animal Ethics Committee.

## Author contributions

FA, SH, MS and CL performed experiments. FA, SH, MS, AW and CL analyzed the results and prepared figures. AW and CL designed the research. FA and AW wrote the paper, which was read and approved by all authors. All authors contributed to the article and approved the submitted version.

## Funding

The authors recognize the support of funding from IMPACT at Deakin University. FA was supported by the Higher Committee for Education Development in Iraq (HCED) and both SH and MS by Deakin University International Postgraduate Research Awards.

## Acknowledgments

The authors would like to thank the Deakin University Animal House staff.

## Conflict of interest

The authors declare that the research was conducted in the absence of any commercial or financial relationships that could be construed as a potential conflict of interest.

## Publisher’s note

All claims expressed in this article are solely those of the authors and do not necessarily represent those of their affiliated organizations, or those of the publisher, the editors and the reviewers. Any product that may be evaluated in this article, or claim that may be made by its manufacturer, is not guaranteed or endorsed by the publisher.
