# Supplementary material for: B cell lymphoma 6A regulates immune development and function in zebrafish
Source: Front Cell Infect Microbiol. 2022 Oct 28;12:887278. doi: 10.3389/fcimb.2022.887278 (PMC9650189; doi:10.3389/fcimb.2022.887278)
Supplement: Supplementary file 1 [file DataSheet_1.pdf]

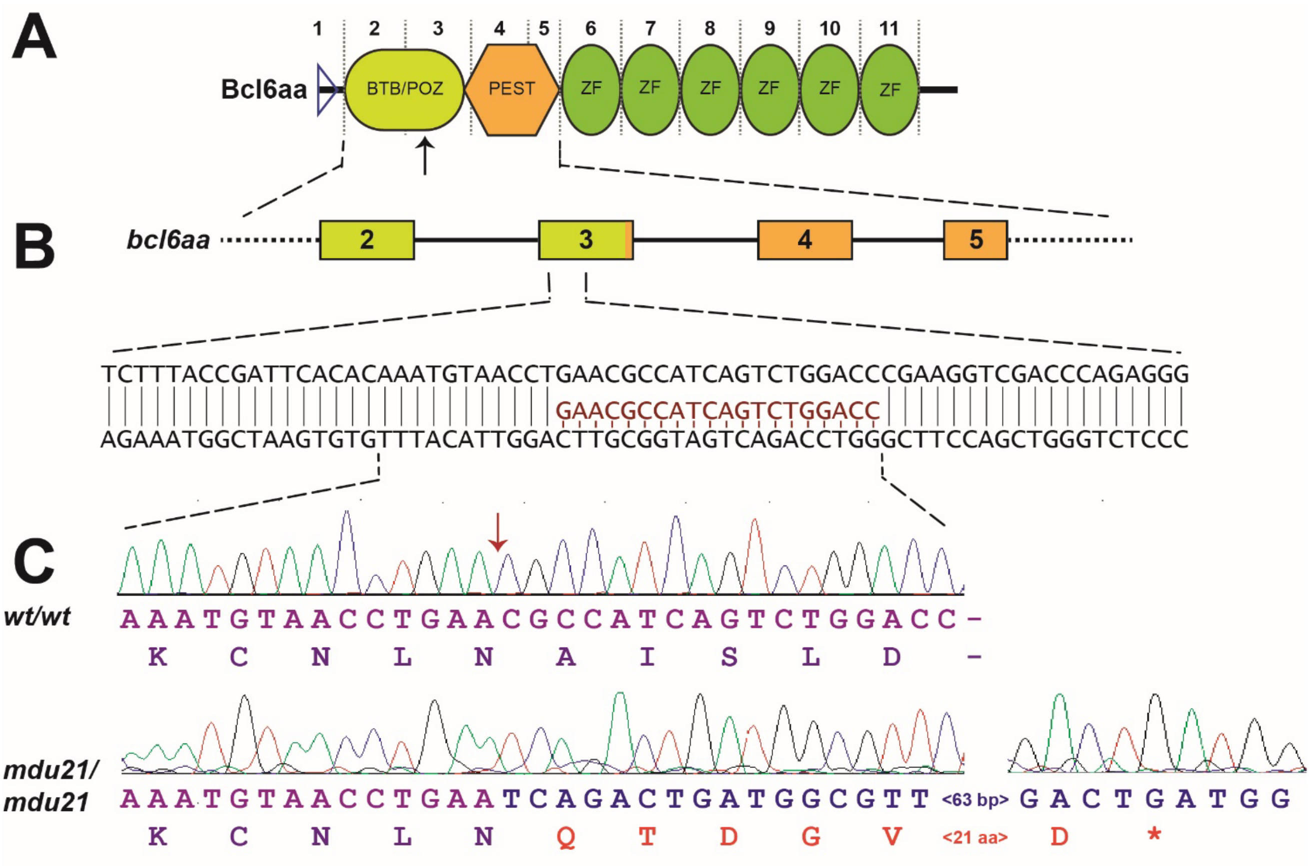

Supplementary Figure 1. Targeting zebrafish *bcl6aa* with CRISPR/Cas9.
A. Schematic representation of zebrafish Bcl6aa protein showing its constituent domains, including the targeted BTB/POZ domain (light green), PEST domain (orange) and ZF domain (dark green). The relative positions of intron/exon boundaries are indicated with vertical dotted lines and exon numbers, with the targeting site designated with a black arrow.
B. Schematic diagram of exons 2-5 of the zebrafish *bcl6aa* gene including an expanded view of the sequence of exon 3 targeted by CRISPR/Cas9 with the gRNA sequence shown in red.
C. Sequence chromatograms and corresponding nucleotide and encoded amino acid sequences obtained from homozygous $bcl6aa^{wt/wt}$ (*wt/wt*) and $bcl6aa^{mdu21/mdu21}$ (*mdu21/mdu21*) individuals. The red arrow indicates the position where *de novo* nucleotides (blue) were inserted into the original sequence (purple) in the mutant to mediate divergence of the encoded protein, with the alternate residues shown in red.

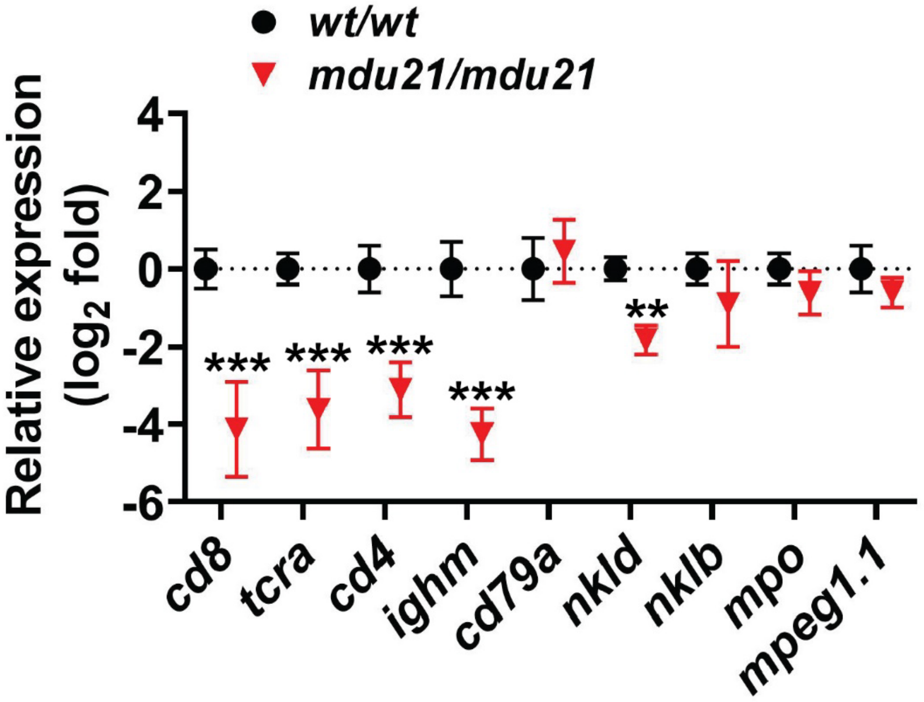

Supplementary Figure 2. Analysis of hematopoietic markers in 28 dpf juvenile zebrafish *bcl6aa* mutants.
Total RNA derived from homozygous *bcl6aa*$^{wt/wt}$ (*wt/wt*) and *bcl6aa*$^{mdu21/mdu21}$ (*mdu21/mdu21*) individuals was subjected to qRT^2-PCR with gene markers of T cells (*cd8*, *tcra*, *cd4*), B cells (*ighm*, *cd79a*), NK cells (*nkld*, *nklb*), neutrophils (*mpo*) and monocyte/macrophages (*mpeg1.*1) (B). Data was normalized relative to *actb* and represented as relative fold change compared to wild-type fish, with mean and SD shown and statistical significance indicated (**: p<0.01, ***: p<0.001, n=6).
